# Fatigue: It Is Not Always in the Head

**DOI:** 10.7759/cureus.39959

**Published:** 2023-06-04

**Authors:** Jehan Zeb, Sana Zafar, Zehra Irshad

**Affiliations:** 1 Acute Medicine, University Hospital Coventry and Warwickshire, Coventry, GBR; 2 Acute Internal Medicine, University Hospitals of Coventry and Warwickshire, NHS Trust, Coventry, GBR; 3 Endocrinology and Diabetes, University Hospital Coventry and Warwickshire, Coventry, GBR

**Keywords:** fast track gca pathway, classification criteria gca, weight loss, giant cell arteritis, fatigue

## Abstract

Fatigue is a common presenting complaint in patients seen in clinics and same-day emergency care. Although it has a simple presentation, it can be challenging to diagnose and manage, particularly when an underlying medical condition presents atypically as fatigue. Here we present an interesting case of giant cell arteritis (GCA) with only fatigue as the presenting complaint. GCA is the inflammation of medium and large vessels in the body, including the aortic arch and its branches. It typically manifests above the age of 50 with headaches, jaw claudication, temporal tenderness, arthralgia, night sweats, and unintentional weight loss. Early diagnosis and treatment are of paramount importance to prevent complications, particularly permanent blindness.

## Introduction

Fatigue is a common presenting complaint seen in primary and secondary care. The prevalence of fatigue has been reported to be up to 50% in patients suffering from chronic illnesses. Multiple other studies across the world have reported the prevalence to be 21.9% (Swiss cross-sectional survey) and 8.2% (Canadian comparative primary care records) [1]. A survey conducted across US primary care stated that seven million visits to general practise per year were due to complaints of fatigue. The prevalence in the population remained between 4.3% and 21.9% [2,3].

Although fatigue is linked to a variety of pathological conditions, it can be easily ignored or missed. In recent years, fatigue has also been seen as part of vasculitis, particularly giant cell arteritis (GCA). GCA is inflammation involving the medium- to large-sized arteries, particularly the aorta and its branches. It typically presents with headaches, jaw claudication, temporal tenderness, arthralgia, night sweats, and weight loss [4]. A United Kingdom (UK) qualitative study showed fatigue as one of the manifestations of GCA, as 82% of the patients diagnosed with GCA reported a Multi-dimensional Fatigue Inventory score (MFI score > 13) [5]. It usually presents along with other symptoms of GCA, and hence the diagnosis is based on the common presentations of GCA rather than fatigue itself. The psychosocial origin of fatigue has been suggested in patients with GCA; however, clear data and evidence bases are not available to support that [4].

In the United Kingdom (UK), giant cell arteritis is the most prevalent vasculitis, with approximately 22 per 100,000 people over the age of 50 being affected each year [6]. The diagnosis of GCA is based on clinical presentation and ACR classification criteria [7]. Timely diagnosis and management of GCA are crucial, as permanent visual loss is one of the most feared complications; in some cases, it could be the initial presentation. In addition, stroke, aortitis, and a consequent aneurysm can also occur if left untreated. Therefore, it is important that typical and atypical symptoms are recognised early to ensure timely management [7].

Here we describe a case of a patient with GCA who presented mainly with fatigue.

This article was previously presented as a poster at the 2020 Society of Acute Medicine, Glasgow conference held on November 12-13, 2020.

## Case presentation

A 71-year-old gentleman was referred by a general practitioner to the medical decision unit (MDU) with a four-week history of fatigue. He complained of generalised weakness in his legs, particularly in his thighs, which led to a few near-fall experiences. He denied any infection symptoms, visual disturbances, headache, shortness of breath, chest pain, arthralgia, trauma, neck stiffness, or jaw claudication. He also complained of reduced appetite, weight loss, and night sweats, which he put down to his tiredness. He had a background of prostate carcinoma (T2b N0 M0) treated with radiotherapy back in 2019, followed by a year of luteinizing hormone-releasing hormone (LHRH) receptor agonist therapy completed in October 2020. He lived alone and was previously mobile independently.

On examination, he looked exhausted and pale. There was no lymphadenopathy or temporal tenderness. His laboratory investigation showed raised inflammatory markers (CRP 150) with an ESR of 113 (Table 1).

**Table 1:** Laboratory investigations WBC: white blood cells, CRP: C-reactive protein, TSH: thyroid-stimulating hormone, ESR: erythrocyte sedimentation ratio, SPEP: serum protein electrophoresis

Investigation	Results	Normal range
Haemoglobin	118 g/L	130–170 g/L
WBC	14.92 × 10^9^/L	4–11 × 10^9^/L
Platelets	462 × 10^12^/L	450–530 × 10^12^/L
CRP	150 mg/L	<5 mg/L
ESR	113 mm/hour	0–30 mm/hour
Peripheral blood film review	Mature monocytosis	
Ferritin	2818 mcg/L	15–350 mcg/L
Vitamin B12	776 ng/L	191–663 ng/L
Folate	6.0 mcg/L	3.3–19.3 mcg/L
TSH	2.38 mU/L	0.27–4.20 mU/L
SPEP	No obvious paraprotein detected.	
Free kappa chains	61.63 mg/L	3.3–19.40 mg/L
Free lambda chains	40.57 mg/L	5.70–26.30 mg/L

**Figure 1:**
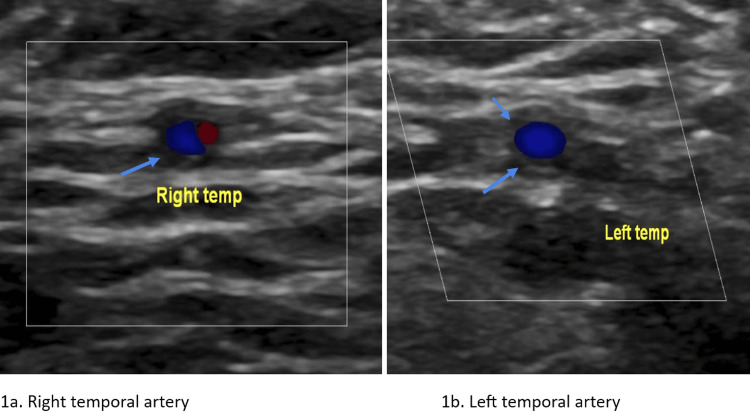
Temporal artery doppler (right and left) showing halo sign

In the context of his prostate cancer and longstanding anaemia, he was also screened for multiple myeloma, which showed significantly raised free light chains and ferritin. The case was also discussed with the haematology team, and they advised proceeding with investigations for underlying infection or inflammation. His infection screen, including hepatitis B, hepatitis C, syphilis serology, and HIV, was negative. Also, CT of the thorax, abdomen, and pelvis was reported as normal. He was also screened for vasculitis (blood autoantibodies including rheumatoid factor (RA factor), antinuclear antibodies (ANA), anti-double-stranded DNA (anti-ds DNA), proteinase-3 antibodies (PR3), myeloperoxidase antibodies (MPO), and complement levels, which were unremarkable. He was then discussed with rheumatology, who advised temporal artery doppler, which showed bilateral temporal artery wall thickening (Halo sign) suggestive of temporal arteritis (Figure 1).

Upon further questioning, he mentioned that he had noticed a mild blurring of vision in the left eye with no diplopia. He had first noted this one month ago; however, since it was only a subtle change in vision peripherally, he did not consider getting any medical advice. Following this, an urgent ophthalmology assessment was arranged, which showed blurring of the medial margin of the left optic disc and left eye supero-temporal visual field loss. The colour vision was normal in both eyes; however, the responses were slower in the left. He was managed with intravenous methylprednisolone 500 mg for three days, followed by oral prednisolone 60 mg once daily for four weeks, with gradual tapering over the course of six months.

Follow up

He was followed up by ophthalmology and rheumatology teams over the course of months. He reported significant improvement in the symptoms of fatigue, with no further visual symptoms. The steroid dose was gradually tapered down, with no further recurrence of the symptoms. The optic disc swelling also improved; however, the supero-temporal visual field loss was permanent.

## Discussion

Fatigue is a common presentation in both primary and secondary care. It can have an impact on the personal and professional lives of people and, hence, result in severe morbidity. Several physical and pathological conditions manifest fatigue as the only symptom; hence, the diagnosis could be difficult to establish with certainty. While investigating for this, multi-system investigations are done most of the time; however, the available literature has suggested that history and physical examination still hold prime importance in diagnosing the aetiology of fatigue [8].

Giant cell arteritis has a broad range of clinical presentations, with new-onset headaches being the most common in people over the age of 50. Ophthalmological involvement is one of the most feared complications of temporal arteritis. The clinical spectrum of presentation ranges from eye pain to amaurosis fugax and ischemic optic neuritis. If the treatment is not started at an earlier stage, this can lead to permanent visual loss. Also, it has been suggested that the elderly population tends to present more with visual symptoms than patients who present earlier in life [7].

In a prospective study conducted over 20 years, 18 of the 85 patients presented with only visual symptoms and no systemic upset. Of the 18 patients, all of them (100%) had varying degrees of visual loss; six (33.33%) had amaurosis fugax; two had diplopia (11.1%); and one presented with eye pain. The identified pathology was anterior ischemic optic neuropathy in 17 patients and central retinal artery occlusion in two patients, which was confirmed by fluorescein angiography. However, the onset of vision loss can be subtle, so in some cases, patients are not aware of the visual involvement until both eyes have been involved. In one case report, the patient remained asymptomatic until both eyes were involved. If left untreated, opposite-eye involvement has been commonly observed [9]. In this case, as well, the patient only reported mildly blurred vision, which he did not deem important to report.

Although studies have reported fatigue as an associated symptom of GCA [10], our case is unique as he did not experience any headache at all, and the only notable symptom of the patient was the complaint of fatigue.

## Conclusions

Fatigue is a significant and potentially vague symptom that requires thorough clinical assessment and investigation to exclude underlying organic medical causes. Rheumatologic conditions like GCA can have a heterogenous clinical presentation and tend not to follow a specific clinical presentation. In cases of diagnostic uncertainty for symptoms like fatigue, uncommon causes of common medical conditions should be investigated, and early specialist input can be helpful for accurate diagnosis and management.
